# Chronic granulomatous disease: a review of the infectious and inflammatory complications

**DOI:** 10.1186/1476-7961-9-10

**Published:** 2011-05-31

**Authors:** EunKyung Song, Gayatri Bala Jaishankar, Hana Saleh, Warit Jithpratuck, Ryan Sahni, Guha Krishnaswamy

**Affiliations:** 1 Department of Pediatrics, Division of Allergy and Clinical Immunology, Quillen College of Medicine, East Tennessee State University, USA; 2 James. H. Quillen VA Medical Center, Johnson City, Tennessee, USA; 3 Department of Internal Medicine, Division of Allergy and Clinical Immunology, Quillen College of Medicine, East Tennessee State University, USA

## Abstract

Chronic Granulomatous Disease is the most commonly encountered immunodeficiency involving the phagocyte, and is characterized by repeated infections with bacterial and fungal pathogens, as well as the formation of granulomas in tissue. The disease is the result of a disorder of the NADPH oxidase system, culminating in an inability of the phagocyte to generate superoxide, leading to the defective killing of pathogenic organisms. This can lead to infections with *Staphylococcus aureus*, *Psedomonas *species, *Nocardia *species, and fungi (such as *Aspergillus *species and *Candida albicans*). Involvement of vital or large organs can contribute to morbidity and/or mortality in the affected patients. Major advances have occurred in the diagnosis and treatment of this disease, with the potential for gene therapy or stem cell transplantation looming on the horizon.

## Introduction

Primary immune deficiencies often present as recurrent infections, often with unusual pathogens, or infections of unusual severity or frequency. Host defense consists of either nonspecific or specific mechanisms of immunity to invading pathogens (Table 1). Nonspecific mechanisms include barrier functions of skin and mucosa (tears, saliva, mucosal secretions, mucociliary responses, and clearance by peristalsis as occurs in the bladder or the gastro-intestinal system), phagocyte responses (neutrophils, macrophages or mononuclear cells and their component of pathogen recognition receptors or PRR and cell adhesion molecules or CAMs), complement system of proteins, C reactive protein (CRP), cytokines, and the PRRs mentioned earlier on the surface of a variety of cell types. Specific immune responses include immunoglobulin class switching and secretion by B lymphocytes/plasma cells and T lymphocyte responses (including antigen recognition, clonal proliferation, and cytokine synthesis resulting in B cell and phagocyte activation and survival).

**Table 1:** Host Immune Defense Mechanisms

Non-Specific	Specific
**Barriers**	Humoral* (Antibodies)
Skin	Cellular* (Lymphocytes)
Secretions (mucous, tears, saliva)	
Mucociliary clearance	
Peristalsis	
**Phagocytes**	
**Complement**	
**CRP and others**	
**Cytokines**	
Pathogen recognition receptors*	

*Major components affected in primary immune deficiency

Innate immunity occurs rapidly and is relatively nonspecific, while adaptive responses occur later and are characterized by activation of the T and B lymphocytes, antigen recognition, cognate interaction using several key cell surface receptors (discussed later) and the synthesis and secretion of antibodies, cytokines and other effector molecules that lead to an expansion of the specific immune response towards a pathogen (Table 2). There are close interactions between the nonspecific/innate and adaptive immune responses, such that a two-way response as well as independent responses regulate overall immune function. To further add a complex dimension to this process is the recent discovery and description of several regulatory T cell subsets including the T regulatory (T reg/Tr1 and Th_3 _subset) and the Th_17 _subset of lymphocytes that oversee overall immune function. The description of these aspects is beyond the scope of this review but needs to be mentioned in order to understand phagocyte function and defects in CGD.

**Table 2:** Innate and Adaptive Immunity

Feature	Innate	Adaptive
**Action Time**	Early (hours)	Late (days)
**Cells**	Macrophage, DC, PMN, NK cells	B and T cells
**Receptors**	TLR: Fixed in genome	Gene rearrangement BCRA, TCR
**Recognition**	Conserved molecules/PAMP	Wide Variety
**Evolution**	Conserved	Only vertebrates

TLR = toll-like receptors; BCRA = B cell receptor for antigen; DC = dendritic cells

PMN = polymorphonuclear leukocytes; B = B lymphocyte; T = T lymphocyte; TCR = T cell receptor for antigen; PAMP = pathogen-associated molecular patterns; NK cells = natural killer cells

**Figure 1:**
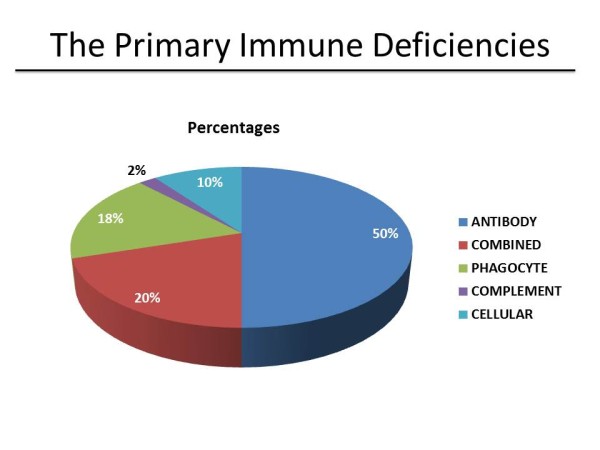
**Distribution of Common Immunodeficiencies**. Primary immune deficiencies can involve either the adaptive (T- and B-lymphocyte deficiencies) or the innate (phagocyte, complement or other defects) immune response (Figure 1). Of these, defects in phagocyte function constitute only about 18%, with the larger portion of the defects seen in the B cell/antibody and/or T cell components of immunity.

**Figure 2:**
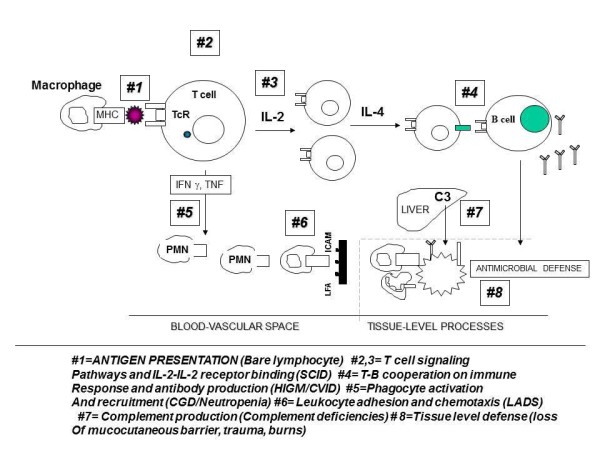
**Defects Leading to Immunodeficiency Disease**. The various defects are summarized in Figure 2, which describe the potential sequence of events, starting from T- and B cell interactions and antibody synthesis to the involvement of phagocyte and complement components of the innate immune response. Explanation for the various aspects of the immune response is provided at the bottom of the figure (components 1-8); Components 5 and 6 deal with phagocytic/neutrophil defects.

Primary immune deficiencies can involve either the adaptive (T- and B-lymphocyte deficiencies) or the innate (phagocyte, complement, or other defects) immune response (Table 2). Of these, defects in phagocyte function constitute only about 18%, with the larger portion of the defects seen in the B cell/antibody and/or T cell components of immunity (Figure 1). These various defects are summarized in Figure 2, which describes the potential sequence of events, starting from T- and B cell interactions and antibody synthesis to the involvement of phagocyte and complement components of the innate immune response. Based on the location of a given immune defect, susceptibility to a particular set of pathogens is likely to occur, which can be explained on the basis of the dominant immune response to any specific pathogen. These aspects are summarized in Table 3.

**Table 3:** Pathogen Patterns in Immune Deficiency

Pathogen Type	T-cell Defect	B-cell Defect	Phagocyte Defect	Complement Defect
**Bacteria**	Bacterial sepsis	Pneumococcus,	Staphylococcus	Neisseria
		Haemophilus	Pseudomonas	Pyogenic bacteria
		*M. catarrhalis*		
**Virus**	CMV, EBV, VZ	CEMA	---	---
**Fungi**	Candida/PCP	PCP	Candida, Aspergillus	---
**Parasite**	---	Giardia	---	---
**Acid Fast**	AFB	---	Nocardia	---

CMV = Cytomegalovirus; EBV = Epstein-Barr virus; VZ = Varicella Zoster;

PCP = Pneumocystis Carinii; AFB = Acid-fast bacillus;

CEMA = Chronic Echovirus Meningoencephalitis of Agammaglobulinemia

M. catarrhalis = Moraxella catarrhalis

Disorders of the innate immune system involve defects in complement as well as defective phagocyte responses to infectious illness. In the latter group are a host of disorders, which are summarized in Table 4. These include chronic granulomatous disease (CGD), neutrophil adhesion defects (such as the leukocyte adhesion deficiency syndromes), Chediak-Higashi syndrome, Griscelli syndrome, Kostmann's syndrome, WHIM syndrome (disorder characterized by myelokathexis), mannose binding lectin deficiency (MBL deficiency), and enzymatic defects within phagocytes such as deficiencies of glucose-6-phosphate dehydrogenase (G6PD), glutathione reductase, glutathione synthetase, and myeloperoxidase. CGD is the most commonly encountered disorder of phagocytes, and is characterized be repeated infections with bacterial and fungal pathogens, as well as the formation of granulomas in tissue. The disease is a disorder of the NADPH oxidase system, culminating in an inability of the phagocyte to generate superoxide, leading to the defective killing of pathogenic organisms. As shown in Table 3, defects in phagocyte function lead to infections with *Staphylococcus aureus*, *Psedomonas *species, *Nocardia *species and fungi (such as *Aspergillus *species and *Candida albicans*). Due to involvement of vital or large organs, such infections can lead to significant morbidity and/or mortality in the affected patients.

**Table 4:** Disorders of the Phagocyte Resulting in Immune Deficiency

• Chronic granulomatous disease (CGD)
• Neutrophil adhesion defects (such as the leukocyte adhesion deficiency syndromes),
• Chediak-Higashi syndrome
• Griscelli syndrome
• Kostmann's syndrome
• WHIM syndrome (disorder characterized by myelokathexis)
• Mannose binding lectin deficiency (MBL deficiency)
• Enzymatic defects within phagocytes- deficiencies of:
○ Glucose-6-phosphate dehydrogenase (G6PD)
○ Glutathione reductase, glutathione synthetase
○ Myeloperoxidase

The following sections discuss the immunobiology of phagocytes, the defects in CGD and the resulting clinical spectrum observed.

## Normal Phagocyte Physiology

**Figure 3:**
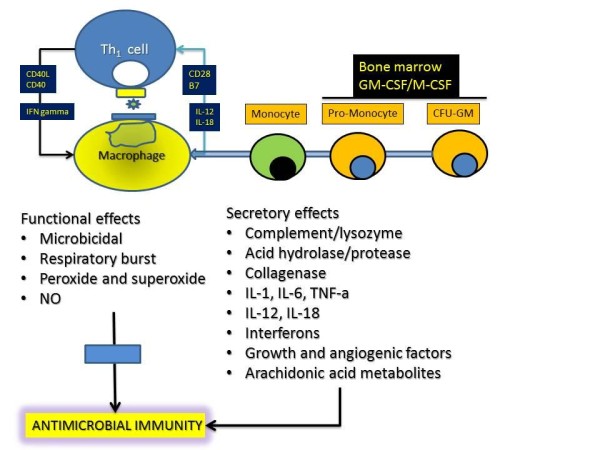
**Role of Macrophages in Host Defenses**. Macrophages are generated from bone marrow precursor cells in the presence of stem cell hematopoietic factors (such as granulocyte-macrophage colony stimulating factor/GM-CSF and macrophage colony stimulating factor/M-CSF). The activation of macrophages (in the presence of specific receptors and T lymphocytes as shown in the figure- CD40/CD40L, CD28/B7, interleukin-interleukin receptor etc) results in functional consequences such as microbicidal activity directed especially towards intracellular pathogens and aided by the expression of peroxides and superoxide radicals. These processes are defective in CGD (see text). Other secretory effects of macrophages result in the production of a plethora of mediators that assist in immunity (these are shown in the figure and include complement, enzymes, interleukins, interferons and growth factors).

**Figure 4:**
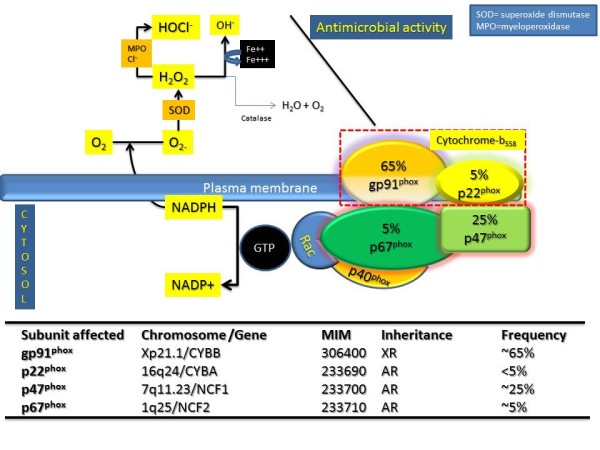
**The NADPH Oxidase System**. The assembly of the various subunits of NADPH oxidase is shown in the figure, while the molecular genetics, rough prevalence, inheritance pattern and chromosomal localization of CGD subtypes are shown in the bottom of the figure. There are several components of NADPH oxidase: of these the cytochrome-b_558 _heterodimer is located in the membrane and consists of the gp91^phox ^and p22^phox ^units, while three cytosolic components exist- including the p67^phox ^, p47^phox ^and a p40^phox^. Following cellular activation, the soluble cytosolic components, p67^phox ^, p47^phox ^, and a p40^phox ^, move to the membrane and bind to components of the cytochrome-b_558 _heterodimer. This is also accompanied by the binding of the GTPase protein, Rac, culminating by unclear mechanisms in flavocytochrome activation. This catalyzes the transfer of electrons from NADPH to oxygen, resulting in the formation of superoxide in the extracellular compartment (phagolysosome). Subsequent reactions via superoxide dismutase (SOD), catalase or myeloperoxidase (MPO), occurring in the phagolysome, can result in formation of H_2_O_2_, H_2_O or HOCl^- ^respectively.

Phagocytes include the neutrophils, monocytes, and macrophages. Much of the early understanding of phagocyte biology resulted from the work of Paul Ehrlich and Metchnikov, pioneers who developed staining techniques and methods to study these cells in vitro. The term phagocytosis was probably introduced by Metchnikov. Hematopoietic growth factors such as GM-CSF and M-CSF regulate phagocyte production from the bone marrow, allowing the development of the monocyte-macrophage lineage cell from the CFU-GM, shown in Figure 3. The macrophage serves pleiotropic roles in the immune response, including presenting microbial antigen to the T cells in the context of major histocompatibility complex, a phenomenon referred to as haplotype restriction. T cells, especially the Th_1 _type cells, secrete interferon gamma (IFN *γ*) which activates macrophages, which in turn express IL-12 and IL-18, thereby allowing the proliferation of Th_1 _lymphocytes. Macrophage activation results in the activation of several processes, aided partly by cognate T-macrophage interaction and also by the PRRs such as the toll-like receptors. The activation of macrophages results in functional consequences such as microbicidal activity directed especially towards intracellular pathogens, and aided by the expression of peroxides and superoxide radicals. These processes are defective in CGD as will be discussed further. Other secretory effects of macrophages result in the production of a plethora of mediators that assist in immunity and are shown in Figure 3. Several of these are also essential aspects of the antimicrobial function of the macrophages. The NADPH oxidase system is pivotal to the anti-microbial function of the phagocyte (neutrophil or macrophage) and its components need to be discussed in some detail in order to understand the molecular pathogenesis and classification of CGD. These aspects are shown in Figure 4 and are discussed later.

## Mechanisms Involved in CGD

CGD represents a heterogeneous group of disorders characterized by defective generation of a respiratory burst in human phagocytes (neutrophils, mononuclear cells, macrophages, and eosinophils). The resultant defect is an inability to generate superoxide and hence an inability to contain certain infectious pathogens. The disease manifests as repeated, severe bacterial and fungal infections resulting in the formation of inflammatory granulomas. The earliest report of the disease was in 1954 by Janeway and colleagues [1]. Landing and Shirkey [2] subsequently described a patient with recurrent infection and associated histiocyte infiltration. A few years later, Bridges and Good reported on a fatal granulomatous disorder in boys and described this as a "new syndrome" [3]. Since these early descriptions, a rather amazing and accelerated understanding of the disease and its molecular genetics have developed in the last 5 decades [4]. Several research groups reported on defective oxidative burst as a possible mechanism of the disease [5-8]. In 1966, Holmes and colleagues recognized an abnormality of phagocyte function in the disease [9], while in 1967 Quie and coworkers demonstrated defective in vitro killing of bacteria by phagocytes obtained from patients with CGD [10]. In that same year, Baehner and Nathan described defective reduction of nitroblue tetrazolium by phagocytes from patients with CGD during phagocytosis, and postulated this as a diagnostic test [11]. This was a seminal paper that led to the critical test used to screen for the disease for decades [12]. This has now been replaced by a flow cytometric assay for the oxidative burst. In 1968, Baehner and Karnovsky demonstrated a deficiency of reduced nicotinamide-adenine dinucleotide oxidase in patients with CGD [13]. This was followed by the demonstration of defective superoxide generation from the phagocytes of patients with CGD by Curnette et al., [14] and confirmation of defective NADPH oxidase expression in the disease by Hohn and Lehrer [15]. In 1986, Baehner reported on the gene localization for X-linked CGD to xp21 [16,17]. Subsequently, defects in several components of the NADPH oxidase complex, including the gp91^phox^, p22 ^phox ^[18], p67^phox ^[18], and p47^phox ^[19] were described by various researchers.

The presumed mechanism of CGD and the associated defects in NADPH oxidase system are shown in Figure 4. The assembly of the various subunits of NADPH oxidase is shown in the figure, while the molecular genetics, rough prevalence, inheritance pattern, and chromosomal localization are shown in the bottom of the figure. There are several components of NADPH oxidase: of these the cytochrome-b_558 _heterodimer is located in the membrane and consists of the gp91^phox ^and p22^phox ^units [4,20,21], while three cytosolic components exist- including the p67^phox ^, p47^phox ^, and a p40^phox^. Following cellular activation, the soluble cytosolic components, p67^phox, ^p47^phox^, and a p40^phox^, move to the membrane and bind to components of the cytochrome-b_558 _heterodimer. This is also accompanied by the binding of the GTPase protein, Rac, culminating by unclear mechanisms in flavocytochrome activation. This catalyzes the transfer of electrons from NADPH to oxygen, resulting in the formation of superoxide in the extracellular component as shown in Figure 4. Subsequent reactions via superoxide dismutase (SOD), catalase or myeloperoxidase (MPO), occurring in the phagolysome, can result in formation of H_2_O_2_, H_2_O, or HOCl^- ^respectively. Recent data suggest that the formation of superoxides and reactive oxygen species is not the end-all of these reactions, as such mediators may also set off subsequent activation of granule proteins such as cathepsin G and elastase, leading to further elaboration of the immune-inflammatory response, all of which are therefore likely to be defective in CGD.

## Molecular subtypes and Genetics of CGD

X-linked CGD (XL-CGD) arises due to mutations in the gp91^phox ^gene and is responsible for 65-70% of the clinical cases in the United States [17,22-24]. This gene is termed CYBB and spans a 30 kb region in the Xp21.1 region (Figure 4). Deletions, frameshift, missense, nonsense, and splice site mutations have been described in this gene. When larger X-chromosomal deletions including the XK gene occur, this may result in a so-called "Contiguous gene syndrome". This may result in associations of the Kell phenotype/Mcleod syndrome with X-linked chronic granulomatous disease (CGD; OMIM 306400), Duchenne muscular dystrophy (DMD; OMIM 310200), and X-linked retinitis pigmentosa (RP3; OMIM 300389)[25,26].

Autosomal recessive CGD (AR-CGD), seen in the remaining 35% cases, arise due to mutations of the other components of the NADH oxidase (except p40^phox ^and Rac which are yet to be associated with any CGD phenotype) [22]: these include- p22^phox ^, p67^phox ^and p47^phox ^. Of these, the dominant mutations observed is of the p22^phox ^gene which accounts for almost 25% cases. The chromosomal location, MIM number, inheritance, and frequency are shown in Figure 4. These phenotypes can be referred to as the X-CGD and as the A22/A47/A67 CGD. Of these subtypes, A47 patients appear to have a less severe course. Nevertheless, extreme heterogeneity may be seen in the manifestations of this disease. Perhaps concomitant immune defects such as those of IgA deficiency or mannose binding lectin mutations (MBL) might be responsible for some of the observed heterogeneity in severity and/or disease progression. These aspects need further study.

## Clinical Features

Patients with CGD usually present in infancy or childhood with repeated, severe bacterial and/or fungal infections. However, delayed diagnosis in adulthood is also possible as is occurrence in females. The disease is relatively uncommon, affecting about 1/250,000 individuals. The most common manifestations include infection, granulomatous disease, inflammation, and failure to thrive (nutritional effects of chronic infection and inflammation). The disease is heterogeneous in its manifestations, related to the subtypes, and severity of the associated macrophage defect [22]. In the majority of patients, the production of superoxides is undetetectable and the manifestations are therefore early and predictable to a great extent. In others, low level respiratory burst activity may delay manifestations or diagnosis into early adulthood [27-30]. Most patients present with infectious illness, which include sinopulmonary disease, abscesses, or lymphadenitis. Other manifestations are related more to inflammatory consequences and/or structural disease and resultant organ dysfunction.

The following sections will discuss the clinical aspects of CGD (X-linked disease and the autosomal recessive disease counterparts), the Kell-deletion/Mcleod syndrome, association with MBL deficiency and other deficiencies and rare clinical manifestations of the carrier state.

## Clinical Aspects

CGD presents most often with infectious illness, though some patients may present with a failure to thrive, granulomatous complications, or inflammatory disease. The disease is usually diagnosed in childhood and sometimes in early adulthood. Table 5 lists the infectious and Table 6 the inflammatory consequences of CGD. Over 90% of the patients with confirmed CGD have severe respiratory burst defects resulting in little or no expression of superoxide radicals. These patients usually present early in life (usually in infancy) as severe or life threatening bacterial or fungal infections. On the other hand, some patients might present in late childhood or early adulthood with recurrent and unusual infections, leading to the diagnosis. Typical infections include purulent bacterial infections (such as pneumonias, sinusitis or liver abscess) or necrotizing fungal infections of deep tissue or bone. As shown in Table 5, common pathogens include the gram negative *Enterobacteriaciae*, *Staphylococcus*, *Nocardia*, *Aspergillus*, *Candida *and atypical *Mycobacteria *[31,32]. Other bacterial include- *Burkholderia *species and *Chromobacterium violaceum*. Many apparent infections go undetected on cultures and may require special efforts to determine a specific etiological organism. At least in Sweden, patients with X-linked disease had more infections than the AR counterparts, with dermal abscesses more commonly seen than lymphadenitis or pneumonias [33]. In the Japanese experience at one hospital, of 23 patients treated, nearly half had Aspergillus infection of the lungs, while short staure and underweight were a complication in up to 1/5^th ^of the patients [34]. Failure to thrive was also observed in the UK series reported [35], where the incidence of growth failure was much higher and listed at 75%. *Aspergillus *as a major cause of morbidity and mortality was also observed in the German cohort [36], where patients with severe involvement of cytochrome b558 were the most likely to manifest complications at an early age and also suffer from more infections compared to those with AR disease. Liese and coworkers described 11 patients with delayed presentations and diagnosis as late as 22 years of age, of which eight had X-linked disease but residual cytochrome function and three had the AR disease, while nine out of the eleven patients had some residual production of reactive oxygen metabolites, explaining their delayed presentation [37]. In a European cohort study consisting of 429 patients [38], 67% had X-linked disease and 33% had the AR counterpart. The patient population consisted of 351 males and 78 females [38]. According to retrospective data collected in this series of patients, AR disease was diagnosed later and the mean survival time was significantly better in these patients (49.6 years) than in XL disease (37.8 years), compatible with other reports from the United States and elsewhere. Pulmonary (66% of patients), dermatological (53%), lymphatic (50%), alimentary (48%), and hepatobiliary (32%) complications were the most frequently observed [38]. *Staphylococcus aureus*, *Aspergillus *spp, and *Salmonella *spp. were the most common cultured pathogens in that order, while *Pseudomonas *spp. and *Burkholderia cepacia *were rarely observed. Roughly 3/4^th ^of the patients received antibiotic prophylaxis, 1/2 antifungal prophylaxis, and 1/3^rd^-received gamma-interferon. Less than 10% of the patients had received stem cell transplantation. Bacterial pneumonia and/or pulmonary abscess, systemic sepsis and brain abscess were the leading causes of death in this series. The differences between the European and United States data/observations are shown in Table 7.

**Table 5:** Infectious Consequences of CGD

Type	Organ System	Manifestations	Etiology	Diagnosis
**Infectious**				
	Blood stream	Sepsis	B Cepacia Pseudomonas Serratia Staphylococcus Salmonella	Blood cultures Echocardiogram
	Pulmonary	Pneumonia	Aspergillus Nocardia Serratia Pseudomonas Staphylococcus Klebsiella Candida Others	Radiolology Cultures Biopsy
	Cutaneous	Impetigo Abscess	Staphylococcus Klebsiella Aspergillus/Candida Serratia	Aspirate cultures Biopsy
	Lymph node	Adenitis Adenopathy	Candida/Nocardia Aspergillus Serratia/Klebsiella	Fine needle aspirate Cultures Biopsy
	Liver	Abscess	Staphylococcus Streptococcus Aspergillus/Nocardia Serratia	Ultrasound or CT Aspirate Biopsy
	Bone	Osteomyelitis	Serratia, Aspegillus Staphylococcus Pseudomonas/Nocardia	Bone scan CT/Biopsy
	GI Tract	Perirectal abscess Fistulae	Enterobacteriaceae Staphylococcus	Biopsy Cultures
	Urinary	Pyelonephritis	Enterobacteriaceae	Cultures IVP/CT etc
				
	CNS	Meningitis Brain abscess	Candida, Haemophilus Aspergillus Staphylococcus	LP, cultures CT/MRI Biopsy

CT = computerized tomography; MRI = magnetic resonance imaging; LP = lumbar puncture; IVP = intravenous pyelogram; CNS = central nervous system; GI = gastrointestinal

**Table 6:** Inflammatory and Structural Complications of CGD

Frequency	Complication
**>50%/Frequent**	Lymphadenopathy
	Hepatosplenomegaly
	Anemia
	Hyperglobulinemia and APR Failure to thrive, underweight
	Failure to thrive, underweight
**≤50%**	Diarrhea
	Gingivitis
	Hydronephrosis
	Gastric outlet obstruction
	Granulomatous ileocolitis
	Stomatitis
	Granulomatous cystitis
	Pulmonary fibrosis
	Esophagitis
	Glomerulonephritis
	Chorioretinitis
	Discoid lupus

**Table 7:** Differences between United States and European Data

Feature	US	European
**Number (n)**	368 (259 XL/81 AR-CGD)	429 (67% XL- and 33% AR-CGD)

**Pneumonia**	79%	66%

**Suppurative adenitis**	53%	50%

**Subcutaneous abscess**	52%	53%

**Liver abscess**	27%	32%

**Osteomyelitis**	25%	NA

**Sepsis**	18%	NA

**Gastric outlet obstruction**	15%	NA

**Urinary tract obstruction**	10%	NA

**Colitis/GI tract**	17%	48%

**Mortality**	18%	NA

XL = x-linked; AR = autosomal recessive

Winkelstein and coworkers reported on the United States CGD experience [30]. Of the 368 patients registered, 259 had the XL-CGD, 81 had AR-CGD, and in the remaining cases the mode of inheritance was unknown. Pneumonia, suppurative adenitis, subcutaneous abscess, liver abscess, osteomyelitis, and sepsis were the most frequently observed complications, in that order (Table 8). Other complications (Table 6) included gastric outlet obstruction, urinary tract obstruction, and granulomatous colitis or enteritis. A small fraction of the XL- and AR-CGD kindreds reported the occurrence of lupus in family members. The most common causes of death were pneumonia and/or sepsis due to *Aspergillus *or *Burkholderia cepacia*. As noted earlier and confirmed in the United States experience, patients with XL-CGD had a more severe phenotype than those with the AR form of the disease.

**Table 8:** Differences between the XL and AR forms of CGD*

Feature	XL CGD	AR CGD
**Family history of lupus**	10%	3%

**Age at diagnosis**	3.01 years	7.81 years

**Perirectal abscess**	17%	7%

**Suppurative adenitis**	59%	32%

**Bacteremia and/or fungemia**	21%	10%

**Gastric outlet obstruction**	19%	5%

**Urinary obstruction**	11%	3%

**Mortality (over 10 year observation)**	21.2%	8.6%

*Adapted from reference 30 (XL = x-linked; AR = autosomal recessive)

### Sinopulmonary Complications

Pneumonia as stated earlier is often the most common complication of the disease. Infections with catalase-positive organisms are the rule. In many cases, no organism is cultured even though the patients are often treated with and respond to antimicrobials directed against bacteria or fungi. The diagnosis of pulmonary involvement is most often made clinically, complemented by radiology (chest roentgenography, computerized tomography or MRI), biopsy, and cultures. Airway obstruction that sometimes complicates infection/granulomatous disease is best diagnosed by pulmonary function tests and by bronchoscopy. Recurrent pneumonia, lung abscess, effusions and empyema thoracis, mediastinal adenopathy, and necrotizing nodular disease may be seen [30]. The common pathogens include *Staphylococcus aureus*, *Burkholderia cepacia*, *Serratia marcescens*, *Nocardia*, and *Aspergillus *spp. In the series reported by Winkelstein et al., pneumonia accounted for 79% of the infectious complications of CGD [30]. Genetically, variant alleles of mannose binding lectin (MBL) were associated with autoimmune disease and may predispose to some pulmonary complications [39]. Chest wall invasion by pathogens has also been described [40] and may be due to necrotizing infections by fungi such as *Aspergillus *[41]. Pulmonary infections have also been described due to *Pneumocystis carinii *[42-44], *Cryptococcus neoformans *[45], *Aspergillus *[41,46-50], visceral *Leishmaniasis *[51], suppurative pathogens [52], *Pseudomonas cepacia *[53,54], [55], *Legionella *[56,57], *Nocardia *[58-61], *Mycoplasma pneumoniae *[62], *Sarcinosporon inkin*-a skin fungus [63], Tuberculosis [64], *Trichosporon pullulans *[65,66], *Tularemia *[67], Q Fever [68], *Acremonium kiliense *[69], *Botryomycosis *[70], *Chrysosporium zonatum *[71], *Burkholderia *(Pseudomonas) *gladioli *[72], fulminant mulch/filamentous fungi [73], Respiratory Syncitial Virus [74], and *Francisella philomiragia *(formerly *Yersinia philomiragia*) [75]. Certain pneumonic variants have also been described in CGD, including crystalline, nodular, and eosinophilic pneumonias [76,77].

### Ocular Complications

Blepharokeratoconjunctivitis, marginal keratitis, and choroido-retinal scars have all been described in CGD [78,79]. A case of congenital arteriovenous hemangioma, presumably related to defective phagocyte function and hemosiderin removal, was described in a patient with CGD [80].

### Neurological Complications

Patients with CGD can develop several neurological complications. Brain abscess has been well described in patients with CGD. Various pathogens have been associated with brain abscess development including *Scedosporium prolificans *[81], *Alternaria infectoria *[82], Salmonella enterica subspecies *houtenae *[83], and *Aspergillus *[84,85]. Other complications associated with CGD include white matter disease [86], CNS granulomatous disease [87] and leptomeningeal, and focal brain infiltration by pigmented, lipid-laden macrophages [88]. Several reports of fungal brain infection [89], *Aspergillus *abscess resembling a brain tumor [90], spinal cord infection by *Aspergillus *[91] and fungal granuloma of the brain have been described [92]. Meningitis due to *Streptococcus *[93] and *Candida *[94] has also been reported on.

### Hepatobiliary and GI Complications

A plethora of GI tract complications occur in CGD. As stated earlier, variant alleles of mannose binding lectin (MBL) were associated with autoimmune disease while polymorphisms of myeloperoxidase and Fc γ RIII were associated more with gastrointestinal complications in patients with CGD [39]. As summarized by Barton et al., GI tract disorders can present from the mouth to the anus, and can be characterized by ulcers, abscesses, fistulae, strictures, and obstructive symptoms [95]. Inflammatory granulomatous colitis can also lead to obstructive disease, diarrhea, malabsorption, or other manifestations [95]. Appendicitis, perirectal abscess, salmonella enteritis, and acalculous cholecystitis have been described, some requiring surgical intervention [96,97].

Gastric outlet obstruction is a recognized complication [98-102]. A case of gastric outlet obstruction due to diffuse gastric infiltration has also been described [103]. There have been reports of nonsurgical resolution of gastric outlet obstruction following the use of glucocorticoids and antibiotics [104]. Liver involvement in the form of hepatic granuloma or multiple hepatic abscesses can complicate management [105-112].

Hepatic involvement by *Staphylococcus aureus *and *Pseudomonas cepacia *can manifest as granuloma or abscess formation [113]. A rare case of ascites has been described in CGD [114] and non-cirrhotic portal hypertension was reported to have prognostic significance [115]. In one study of 194 patients from the NIH, elevated liver enzymes (mainly transaminitis) were documented in >75%, liver abscess in 35%, hepatomegaly in 34%, and splenomegaly in over 50% cases [116]. Liver histology demonstrated granuloma in 75% and lobular hepatitis in 90%. Venopathy of the portal vein was observed in 80% and was associated with splenomegaly [116]. Ament and Ochs commented on the occurrence of several gastrointestinal manifestations in patients with CGD [117,118]- including granulomata on biopsy, malabsorption syndromes, and B12-deficiency. Interferon gamma therapy, careful use of glucocorticoids and liver transplantation have improved outcomes in some patients with liver involvement [119,120]. Chronic infection, nausea, vomiting, and malabsorption can lead to weight loss and/or failure to thrive in patients with CGD [70,95,121-123]. Catch up growth tends to occur and many patients attain predicted heights by late adolescence [124].

### Renal and Gentourinary Complications

Granulomatous involvement and/or infectious complications can result in major genitourinary complications in patients with CGD. Use of glucocorticoids and antimicrobials has resulted in remission of obstructive pathology in some patients, thereby avoiding surgery. Frifeit and coworkers described chronic glomerulonephritis in a 12 year old male with CGD [125]. This culminated in terminal uremia and fatal pulmonary Aspergillosis and *Pseudomonas *septicemia. Diffuse infiltration of renal and other tissues by pigment-containing macrophages may also result in pathology in CGD [126]. Renal Aspergilloses resulting in renal abscess formation has also been described [127] as well as xantogranulomatous pyelonephritis and renal amyloidosis [128,129]. In the latter case, renal amyloidosis resulting in nephritic syndrome occurred in a patient with CGD post-renal transplantation [129]. In one series, 23/60 patients (38%) with CGD demonstrated urological disease [130], including bladder granulomas, urethral strictures, recurrent urinary tract infections, and renal dysfunction. The judicious use of glucocorticoids and interferon gamma has had a beneficial effect on several of these conditions of either the genitourinary or gastrointestinal systems.

### Other Complications

Dermatological manifestations in patients with CGD include atopic dermatitis-like disease but with systemic or deep seated infections [131], facial granulomata [132] and discoid lupus, and seborrheic dermatitis-like disease [133]. Vesicular and granulomatous of fungal skin lesions have been observed in small reports [134]. Altered skin Rebuck window responses in patients with CGD have been recorded [135]. Several skeletal complications related mainly to infections have been described in patients with CGD. Osteomyelitis secondary to invasive *Aspergillus *or *Burkholderia gladioli *[136-140] may occur. Osteomyelitis may involve either the long bones or even the spine [137,139]. Dactylitis may complicate CGD [141]. Multifocal osteomyelitis secondary to *Paecilomyces varioti *has also been reported [142] as has sacral osteomyelitis secondary to *Basidiomycetous *fungi such as *Inonotus tropicalis *[143]. Gill et al., reported on a favorable response to Interferon gamma in osteomyelitis complicating CGD [144]. Occasionally recombinant hematopoietic growth factors (rhG-CSF), long term antimicrobials, and surgery may be required in the management of these complex patients [140,145].

### Inflammatory Responses in CGD

Table 6 lists the inflammatory and structural changes observed in CGD. Some of these changes may truly represent infectious complications, but as stated earlier, many such tissues fail to grow any identifiable pathogens in culture. These changes may also represent the exuberant inflammatory response seen in the disease. Whether these changes represent an overwhelming response to infection by other intact components of the immune response (such as T cells and B cells) and manifested by hyperglobulinemia and elevated acute phase reactants, a failure of the compensatory "anti-inflammatory response" [146], a diminished production of specific regulatory products such as PGE_2 _[147] or activation of nuclear factor kappaB, the ubiquitous transcription fact [148] is unclear. This in addition to the poor superoxide radical response [149,150], failure of phagocytosis and the enhanced cytokine responses [151] may be responsible for the observed inflammatory pathologies listed in Table 6. At least in a murine model, the failure of an immune-modulatory effect of superoxide radicals was associated with exuberant inflammatory responses and TH_17_-mediated pathology and arthritis [152]. The development of animal models of CGD to study this "hyperinflammation" further will improve our understanding of the immune dysregulation seen in the disease [153,154]. Granuloma formation is often accompanied by inflammatory, obstructive, or functional impairments of organ systems, such as the GI tract or the GU system [155].

### Other Aspects of CGD

In some patients with CGD, the deletion in the Xp21 can extend to other "contiguous" genes resulting in an association of the disease with lack of Kell blood antigens (Mcleod phenotype), Retinitis Pigmentosa, and Duchenne Muscular Dystrophy [156-159]. This could complicate blood transfusion. There have been reports of successful granulocyte transfusions in patients with the Mcleod Phenotype, complicated only by mild hemolysis [160].

Female carriers have manifested some symptoms, such as dermatitis, stomatitis, and discoid lupus-like disease [161-165]. CGD-like infections can sometimes present in female carriers, and these patients demonstrate both normal and CGD-neutrophils with functional mosaicism[166]. In one report of 15 carriers of the CGD gene, five patients had both stomatitis and discoid SLE-like lesions and five patients had stomatitis alone, while the remaining five patients were relatively asymptomatic [167]. Martin-Villa and co-workers also described more frequent autoantibodies among carriers of the CGD gene compared to non-carrier relatives, probably related to random X-chromosome inactivation [168].

Concomitant immune deficiencies may complicate CGD and contribute to infectious complications. In patients with documented CGD, variant alleles of mannose binding lectin (MBL) were associated with autoimmune disease and may predispose to some pulmonary complications [39]. There have been several reports of IgA deficiency in patients with CGD [55,169,170]. Further studies of humoral immune response and MBL deficiency in patients with CGD are essential to further understand these immune interactions and predisposition to autoimmunity and infectious disease.

## Diagnosis and Treatment

The approach to the diagnosis of CGD is shown in Table 9. Many of the clinical and laboratory abnormalities suggest the diagnosis. The confirmatory test is measurement of the oxidative burst (superoxide production) of the neutrophil in response to stimulation. While the NBT slide test was commonly used in the past [11,12,171-173], this has been replaced recently by the dihydrorhodamine 123 (DHR) and flow cytometric analysis- the DHR test [174,175]. The DHR test has the ability to distinguish X-linked from the AR forms of the disease as well as pick up the carrier state [176-178]. Other tests proposed include denaturing high-performance liquid chromatography (DHPLC) system [179] and real time PCR-based assays [180,181]. Molecular mutational analysis can help confirm the diagnosis, define the defect, and classify the patient into specific subtypes.

**Table 9:** Approach to Diagnosis of CGD

**Clinical information**
1. Severe, recurrent pulmonary and hepatic infections including abscess formation
2. Specific etiologic pathogens such as B. cepacia, Nocardia, Aspergillus etc
3. Granulomatous lesions of the GI tract or the GU system
**Laboratory abnormalities**
1. Anemia
2. Polyclonal hyperglobulinemia
3. Elevated acute phase reactants such as ESR or CRP
4. Normal studies of T and B lymphocyte immunity
**Diagnostic test**
1. NBT test (no longer used)
2. DHR
**Molecular tests**
1. Immunoblotting or flow cytometry
2. Molecular techniques including gene sequencing and mutational analyses for subtype

NBT = nitroblue tetrazolium slide test; ESR = erythrocyte sedimentation rate; CRP = C reactive protein; GI = gastrointestinal system; GU = genitourinary system

Treatment of CGD includes prophylaxis of infections using interferon gamma and appropriate antimicrobials (such as trimethoprim sulfamethoxazole/TMP-SMX and itraconazole), and management of infections using appropriate antimicrobials based on pathogen likelihood or identification. In select situations, granulocyte transfusions may be required [182-184]. The roles for stem cell transplant (HLA identical sibling umbilical cord stem cell transplantation (UCSCT) after myeloablative conditioning [185-187]) and gene therapy need to be defined [188-192]. Allogeneic hematopoietic stem cell transplantation from a HLA-identical donor may, at present, be the only proven curative treatment for CGD [187]. Summaries of various treatment options are provided in Table 10.

**Table 10:** Treatment of Chronic Granulomatous Disease

Prophylaxis of Infection	
*Antibacterial therapy*	Trimethoprim-sulfamethoxazole (TMP-SMX) 5 mg/kg/day (based upon the TMP component, maximum dose 320 mg P.O in two divided daily doses) [187]
*Antifungal therapy*	Itraconazole 5 mg/kg [85] (maximum dose 200 mg orally daily)
*Immunomodulatory therapy*	Interferon-gamma (IFN-*γ*) [85,137] 50 μg/m^2 ^(subcutaneous) three times a week 1.5 μg/Kg (subcutaneous) three times a week for children <0.5 m^2^
**Management of Infection**	
***Empirical treatment***	TMP-SMX/Fluoroquinolone/Antifungal (Voriconazole)
	• *Burkholderia, Serratia *species: TMP-SMX
	• *Nocardia *species: TMP-SMX and/or Cabapenem
	• *Staphylococcus aureus*:TMP-SMX or Vancomycin
	• Fungal infection: Antifungal agent ±Steroid
***Liver abscess***	Surgical excision [111]; IFN *γ *[108,120]
***Granulocyte Transfusion***	Unirradiated white blood cells [183,184]
**Definitive treatment**	
***Stem cell transplant***	HLA identical sibling umbilical cord stem cell transplantation (UCSCT) after myeloablative conditioning (Stem cell transplantation from a HLA-identical donor may, at present, be the only proven curative approach to CGD) [185-187]
***Gene therapy***	Still experimental [188-192]

## Competing interests

The authors declare that they have no competing interests.

## Authors' contributions

ES- organized manuscript, assisted in writing treatment portion; GJ- organized manuscript, assisted in writing treatment portion; HS- assisted with manuscript review and corrections; WJ- assisted with manuscript review, references and corrections; RS- assisted with manuscript editing, assignment of figures and tables and generation of some of the tables; GK- conceived of the manuscript, generated all figures and developed the format. All authors have read and approved the final manuscript.
